# Modern Rare Earth Imprinted Membranes for the Recovery of Rare Earth Metal Ions from Coal Fly Ash Extracts

**DOI:** 10.3390/ma17133087

**Published:** 2024-06-24

**Authors:** Aleksandra Rybak, Aurelia Rybak, Sławomir Boncel, Anna Kolanowska, Agata Jakóbik-Kolon, Joanna Bok-Badura, Waldemar Kaszuwara

**Affiliations:** 1 Department of Physical Chemistry and Technology of Polymers, Faculty of Chemistry, Silesian University of Technology, 44-100 Gliwice, Poland; 2 Department of Electrical Engineering and Industrial Automation, Faculty of Mining, Safety Engineering and Industrial Automation, Silesian University of Technology, 44-100 Gliwice, Poland; aurelia.rybak@polsl.pl; 3 Department of Organic Chemistry, Bioorganic Chemistry and Biotechnology, Faculty of Chemistry, Silesian University of Technology, 44-100 Gliwice, Poland; slawomir.boncel@polsl.pl; 4 Institute of Chemistry, Faculty of Science and Technology, University of Silesia, 40-007 Katowice, Poland; 5 Department of Inorganic Chemistry, Analytical Chemistry and Electrochemistry, Faculty of Chemistry, Silesian University of Technology, 44-100 Gliwice, Poland; agata.jakobik@polsl.pl (A.J.-K.); joanna.bok-badura@polsl.pl (J.B.-B.); 6 Faculty of Materials Science and Engineering, Warsaw University of Technology, 02-507 Warszawa, Poland; waldemar.kaszuwara@pw.edu.pl

**Keywords:** ion-imprinted polymers, adsorptive materials, REE recovery, coal fly ashes

## Abstract

The need to identify secondary sources of REEs and their recovery has led to the search for new methods and materials. In this study, a novel type of ion-imprinted adsorption membranes based on modified chitosan was synthesized. Their application for the recovery of chosen REEs from synthetic coal fly ash extracts was analyzed. The examined membranes were analyzed in terms of adsorption kinetics, isotherms, selectivity, reuse, and their separation abilities. The experimental data obtained were analyzed with two applications, namely, REE 2.0 and REE_isotherm. It was found that the adsorption of Nd^3+^ and Y^3+^ ions in the obtained membranes took place according to the chemisorption mechanism and was significantly controlled by film diffusion. The binding sites on the adsorbent surface were uniformly distributed; the examined ions showed the features of regular monolayer adsorption; and the adsorbents showed a strong affinity to the REE ions. The high values of K_d_ (900–1472.8 mL/g) demonstrate their high efficiency in the recovery of REEs. After five subsequent adsorption–desorption processes, approximately 85% of the value of one cycle was reached. The synthesized membranes showed a high rejection of the matrix components (Na, Mg, Ca, Al, Fe, and Si) in the extracts of the coal fly ashes, and the retention ratio for these Nd and Y ions was 90.11% and 80.95%, respectively.

## 1. Introduction

Recently, the significance of rare earth metals (REEs) has been growing due to their extraordinary chemical, catalytic, physical, magnetic, and luminescent properties, despite limited availability, and their numerous applications, especially in modern technologies [1,2]. In addition to the use of conventional sources, alternative sources of these elements have been sought, especially in the area of waste material that could become an excellent secondary source, such as coal fly ash. A huge amount of this type of waste material is generated annually (over 750 million tons), yet only 30% is utilized [3].

REEs can be recovered from coal fly ash using biological, physical, and chemical methods [2]. However, the biological and physical methods are less effective [4]. The chemical method, which is the most promising, consists of several steps. In the first stage, during acid–base leaching, the REE ions are carried into a solution and then extracted with reagents. In the case of the recovery of the REE ions from these leachates, problems related to an extreme pH, the high complexity of the obtained solutions, and the high concentration of ash matrix components (Na, Mg, Fe, Si, Al, Ca, etc.) need to be solved [5]. The separation and purification of the individual REEs from the obtained mixtures is also difficult, because of their chemical similarities [2,5,6].

To achieve the recovery, some conventional and innovative techniques have been proposed. The chemical methods of REE recovery include ion exchange, coagulation, flocculation, flotation, adsorption, and chemical precipitation. However, these chemical methods have some limitations, such as high energy consumption, non-selectivity, problems with regeneration, and the application of enormous amounts of chemical reagents, which are associated with high environmental and operational costs [7]. Therefore, alternative methods have been introduced, such as membrane techniques and methods based on chelating compounds and ionic liquids [8,9,10,11,12].

As a result, further research into the separation of chemically similar REE elements from leachates with extreme pHs and complex compositions should develop in the direction of membrane and sorption techniques, based on modern materials with a high selectivity and affinity to REEs. In the case of membrane technologies, their use for the selective recovery of metal ions can only be easily achieved by using appropriate materials like, for instance, ion-imprinted polymers (IIPs), which have recognition sites in the macromolecular matrix made by using a template molecule. IIPs are usually stable, easy to synthesize, REE-selective in the presence of other metal ions, inexpensive, and reusable.

Until now, IIPs have been used either as the solid phase in SPE to preconcentrate Eu, Ce, Nd, Dy, and Y from wastewaters, human serum, and plasma or as a sensor [13,14]. IIPs are cross-linked polymers with structures that are rich in pores and binding sites intended for the targeted ions [15,16]. They can be synthesized in a few steps during a reaction between the functional monomer, crosslinker, initiator, and template. In the first stage, complexes are formed from monomers containing functional groups and template ions (that is, REE ions). In the second step, the monomers are polymerized by adding cross-linking agents and carrying out thermo- or photopolymerization. In the third stage, the template REE ions are removed from the polymer matrix, which leads to the creation of specific binding places that can later capture the considered ions [17].

IIPs have a high ion selectivity due to the presence of these binding sites that can bind ions of the right size and charge. Their adsorption ability depends on several factors, such as the ions’ size, charge, electron configuration, and oxidation degree and the ability of the ligands to bind the ions. These polymers are characterized by the appropriate thermal and pH stability. IIPs were developed to imitate the lock and key mechanism, are used to recognize and remove target ions, and are characterized by a high selectivity. Even trace analytes or pollutants can be selectively removed with the help of IIPs, which has not yet been achieved effectively with other methods [18,19,20,21]. To synthesize these polymers, there were a few methods that were initially based on binary complexes; however, they have become less efficient than those based on ternary complexes. As a result, current research is moving towards the synthesis of IIPs based on ternary complexes, such as during the copolymerization of a complex between REE(III), 5,7-dichloroquinoline-8-ol (DCQ), 4-VP, and some other monomers, with styrene-DVB, HEMA-EDMA, and MMA-EDMA as the monomers and crosslinkers [13,22,23,24,25,26,27,28,29,30]. However, it has been stated that styrene-DVB-based IIPs could be a better choice for the separation of REEs compared to HEMA-EDMA- and MMA-EDMA-based IIPs. Other IIPs exhibiting a strong affinity towards the chosen REE ions were synthesized using the corresponding rare earth materials and a complexing agent as the Schiff base ligand in the presence of EGDMA as the cross-linking agent, 4-vinylpyridine as the functional monomer, and AIBN as the initiator [15,31,32]. However, about 80% of the REE recovery is all that these applied IIPs have been able to obtain [33]. The problem that needs to be solved is the leakage of DCQ (with a negative impact on the recovery of REEs), which, however, can be mitigated by using DMSO in the synthesis instead of 2-methoxyethanol [13].

It should be noted that the introduction of materials based on ion-imprinted polymers will avoid most of the problems faced by researchers using membrane, biological, or chelating compound-based methods. Namely, it was found that in the case of membrane methods, especially those based on NF, ELM, and HFLM, REE enrichment, even above 90%, was achieved [10,11]. However, in the case of problems related to increasing the efficiency of the process and extending the life of the membranes (as opposed to fouling), there is a need to introduce additional stages of the membrane process in the form of a connection with additional MF modules, a complexation stage with EDTA or DTPA (increasing the REE recovery and the removal of Fe, Al, and Si), or the introduction of modern ion-imprinted polymers (with the separation of individual REEs from the mixture) [7,15]. Other methods used to recover the REEs from extracts are biological methods based on bioleaching using microorganisms. These methods are less efficient compared to other techniques, but they are environmentally friendly. The recovery of REEs that can be obtained with their use depends mainly on the form of REE occurrence and the type of microorganisms used, with a recovery rate of over 74% in the cases of Candida bombicola and Aspergillus ficuum, for example [34]. The next method from the group of alternative methods is the use of chelating compounds and ionic liquids. When using chelating compounds such as EDTA, EDDS and HIDS, only 18% of Ce was recovered [35]. In the case of ionic liquids, REE enrichment can reach up to 95%, but only if expensive, appropriate ionic liquids are selected and multi-stage extraction is performed [36].

The aim of this work is the synthesis of Nd- and Y-selective ion-imprinted membranes based on modified chitosan and their application for the recovery of chosen REEs from synthetic coal fly ash extracts. The obtained membranes were examined using various techniques like XRD, SEM, TGA, and VSM. The experimental data were also analyzed by the usage of two applications, namely, REE 2.0 and REE_isotherm.

These two rare earth elements, namely, Nd and Y, were chosen because of their widespread applications, such as in the production of the permanent magnets used in a wide range of electronic devices (computer hard drives, headphones, and speakers) and wind turbines; the electric motors for hybrid and electric vehicles; medical devices like, for instance, MRI machines; the production of color television tubes; LED lighting; medical lasers and laser cutting tools; microwave and radar applications; and as catalysts (e.g., in the production of polymers) to improve the properties of certain metals, such as aluminum and magnesium [1,2,37,38,39,40,41,42].

## 2. Materials and Methods

### 2.1. Reagents

Chitosan, acetic acid, sodium hydroxide, absolute ethanol, o-nitrobenzaldehyde (ON), hydrazine monohydrate, glyoxylic acid, zinc powder, Nd(NO_3_)_3_·6H_2_O, Y(NO_3_)_3_·6H_2_O, glutaraldehyde, and hydrochloric acid were obtained from Merck Life Science (Poznan, Poland). All the reagents were of an analytical grade. Certified single element standard solutions of Nd, Pr, Dy, Gd, and Y with concentrations of 1000 µg/mL and Na, Mg, Ca, Fe, Al, and Si with concentrations of 10,000 µg/mL were obtained from Inorganic Ventures (Christiansburg, VA, USA). Deionized water was used in all the experimental processes.

### 2.2. Membrane Characterization

The synthesized membranes were also characterized by thermogravimetry (TGA), scanning electron microscopy (SEM), vibrating sample magnetometry (VSM), and X-ray diffraction (XRD).

The magnetic properties of the obtained adsorptive membranes were tested using the Lake Shore 7010 vibration magnetometer (VSM) (Westerville, OH, USA).

A Linseis STA PT1600 thermobalance (Selb, Germany) was used for the TGA analysis (under an argon atmosphere of 60 mL min^−1^ and with a heating rate of 10 °C min^−1^). The SIX HITACHI S-3400N SEM (Hitachinaka, Japan) apparatus was used for the scanning electron microscopy analysis of the obtained membranes (at 5 kV). A Rigaku MiniFlex II diffractometer (Tokyo, Japan) with filtered cooper radiation CuKα (λ = 1.54178 Å) was used for this purpose. The diffraction pattern was obtained in a range of 2θ = 10–90° with continuous recording at a speed of 0.4°/min.

### 2.3. Membrane Preparation of the REE IIPs

**Figure 1:**
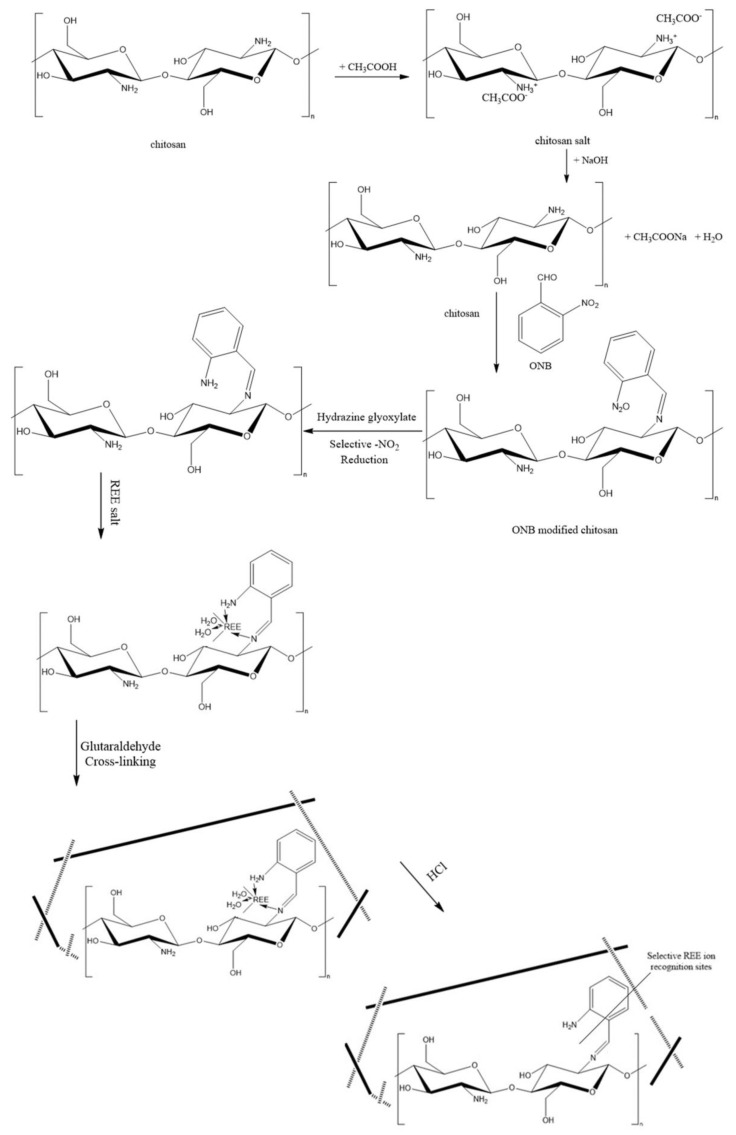
Scheme of the multi-stage reaction for obtaining REE ion-imprinted modified chitosan.

The procedure to obtain IIPs printed with REE ions was based on modified chitosan (Figure 1). It consists of three steps. In the first step, the Schiff base was obtained. To achieve this, 10 g of chitosan was stirred with 500 mL of 1% acetic acid and then was injected into 700 mL of 2% NaOH solution. Then, swollen gelatins were washed with 5 L of deionized water and 300 mL of absolute ethanol to a pH of 7.0. After that, they were transferred into a flask into which 200 mL of 20% o-nitrobenzaldehyde in ethanol was added, and the whole mixture reacted for 12 h at 80 °C under reflux. The product was washed with ethanol. In the second step, the Schiff base was subjected to equal moles of hydrazine hydrate (0.2 mol) and glyoxylic acid (0.2 mol), along with 200 mL of deionized water and 2 g of zinc powder, which were mixed for 60 min at room temperature. After washing with deionized water and ethanol, the modified yellow chitosan was obtained and then dried at 40 °C. In the third step, 20 mL of 4% modified chitosan solution (in 2% acetic acid) was stirred with 0.8 g of REE(III) salt for 12 h and 2 h under the ultrasounds. Then, 0.1 mL of 50% glutaraldehyde solution was added and stirred for 0.5 h. After an additional 15 min under the ultrasounds, the solution was cast on a levelled PTFE Petri dish and dried at 27 °C in a vacuum dryer. Then, the membrane was washed with water before being leached with 1 M of HCl solution to remove the REE ions and to obtain the imprinted cavities. Before carrying out the measurements, the membranes were soaked in 1 M of sodium hydroxide solution and then stored in deionized water.

### 2.4. The Evaluation of the Separation Abilities of the Adsorptive Membranes

**Figure 2:**
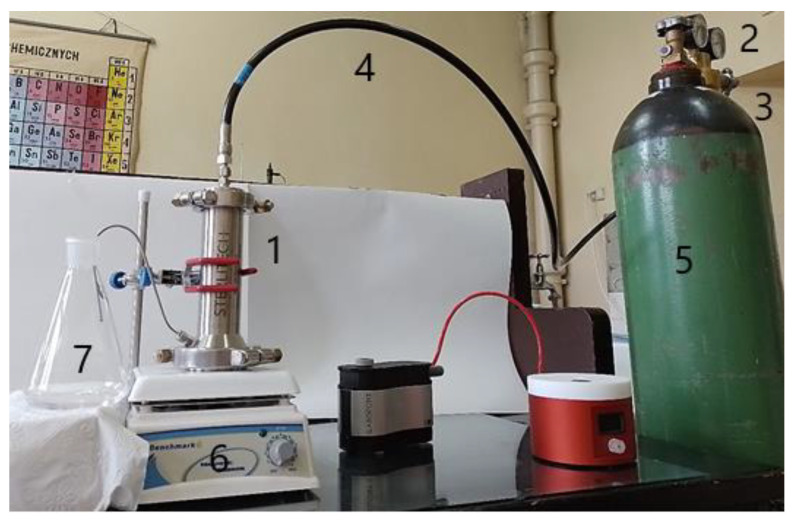
Experimental setup. 1—high-pressure stirred membrane cell, 2—gas regulator, 3—bleed valve, 4—high pressure hose, 5—nitrogen gas cylinder, 6—magnetic stirring plate, 7—permeate collection vessel.

The obtained adsorptive membranes were investigated using a Sterlitech HP4750 (Auburn, WA, USA) high-pressure stirred cell kit. The whole experimental setup consisted of a high-pressure stirred membrane cell, a regulator, a bleed valve, a high-pressure hose, a nitrogen gas cylinder, a magnetic stirring plate, and a permeate vessel (Figure 2).

Before the measurement, the examined membrane (with an active area of 14.6 cm^2^) and PTFE-coated magnetic stir bar mechanism were installed in a high-pressure membrane cell. Then, the vessel was filled with the deionized water, and, after preconditioning with a feed, a solution (200 mL) was separated, the composition of which is shown in Table 1. The stir bar was driven by a magnetic stirring plate to minimize the concentration polarization at the membrane surface. After that, the appropriate pressure (20–60 bar, controlled by means of a regulator) of nitrogen was introduced to the membrane cell by an inlet, and the permeate was collected by the permeate vessel. The entire process was performed at a temperature of 20 °C, with an appropriate pH of 7.5 for the feed solution (which is the optimal value, based on adsorption experiments), and, depending on the analyzed membrane, lasted from 4 to 6 h. The recovery of the adsorbed REE ions was performed in the same setup by the application of 50 mL of 1 M HCl after the separation process. The compositions of the permeate, retentate, and recovery solution were analyzed using the ICP-AES technique. The characteristic retention coefficient R was also calculated for the examined REE ions:

(1) $$R=\left(1-\frac{C_{p}}{C_{r}}\right)\cdot 100\%$$

where *c_p_* denotes the concentration of the REE ion in the permeate [mg/L]; and *c_r_* is the concentration of the REE ion in the retentate [mg/L].

### 2.5. Adsorption Experiments

#### 2.5.1. The Effect of pH on Adsorption

In total, 50 mg of adsorptive material was immersed in 50 mL of 50 mg/L of REE ion solution (neodymium (III) nitrate and yttrium (III) nitrate) with an initial pH in the range of 2 to 9 for 12 h at 25 °C. The final REE concentration was determined by an ICP-AES. The pH was set using the NaOH or HCl addition.

#### 2.5.2. Kinetics Research

To examine the mechanism controlling the REE ion adsorption speed, 50 mg of adsorptive material was immersed in the REE solution (50 mL, 50 mg/L, pH = 7.5, and 25 °C) for a strictly defined contact time. The final concentrations of the REE ions were taken at specified times and were determined by the ICP-AES: 5, 15, 30, 60, 90, 180, 360, and 720 min. The *qt* values were calculated using the following formula:

(2) $$q_{t}=\frac{\left(C_{0}-C_{k}\right)V}{m}$$

where

*V* denotes the volume of the solution [L];
*m* is the mass of the adsorptive material [mg];
*q_t_* is the amount of the REE ions adsorbed at time t [mg/g];
*C*_0_ and *C_K_* are the concentrations at the initial time and time t [mg/L].

#### 2.5.3. Adsorption Isotherm Studies

In total, 50 mg of adsorptive material was immersed in 50 mL of REE solution (pH = 7.5 and 25 °C), whose initial concentrations were 20, 50, 75, 100, 125, 150, 175, and 200 mg/L. The final REE ion concentration was determined by the ICP-AES.

The equilibrium adsorption capacity *q_e_* [mg/g] was calculated according to the following equation:

(3) $$q_{e}=\frac{\left(C_{0}-C_{e}\right)V}{m}$$

where

*C*_0_ and *C_e_* denote the initial and equilibrium concentrations [mg/L];
*V* is the volume of the solution [L];
*m* is the mass of the adsorptive material [mg].

#### 2.5.4. Selectivity Tests

Selectivity tests were performed for the synthetic solutions containing Nd, Pr, Dy, Gd, and Y and the matrix elements Ca, Fe, Al, Na, Mg, and Si. The total volume of the solution was 50 mL with a concentration of 50 mg/L of each metal ion. The pH of the solution was 7.5, which was regulated by the addition of the NaOH solution. In total, 50 mg of the adsorptive material was immersed in this solution and placed into a water bath at 25 °C for 12 h.

The *K_d_* was calculated, i.e., the distribution coefficient, as follows:

(4) $$K_{d}=\frac{\left(C_{0}-{\;C}_{f}\right)V}{C_{f}m}$$

where *C_f_* is the final concentration [mg/L] and the selectivity coefficients are *k*, as follows:

(5) $$k=\frac{K_{d1}}{K_{d2}}$$

where

*K_d_*_1_ is the distribution coefficient of the considered REE ion;
*K_d_*_2_ is the distribution coefficient of the competitive metal ion.

#### 2.5.5. Stripping and Reusability Experiments

After the adsorption process, the adsorbents were extracted from the remaining solution, washed with deionized water, and regenerated with 1 M of HCl at room temperature for 48 h and washed again with deionized water. Then, they were used again in the adsorption process with a repetition of five cycles.

### 2.6. ICP-AES Analysis

The obtained solutions during the adsorption experiments were analyzed using the ICP-AES technique.

The concentrations of the REEs and other elements were measured using the Varian 710-ES inductively coupled plasma atomic emission spectrometer (ICP-AES, Varian, Palo Alto, CA, USA) equipped with a SeaSpray nebulizer and Twister glass spray chamber. The parameters of the analysis were as follows: RF power, 1.0 kW; plasma flow, 15 L/min; auxiliary flow, 1.5 L/min; nebulizer pressure, 200 kPa; pump rate, 15 rpm; and emission wavelengths of Gd 335.048, 335.863, and 342.246 nm; Dy 340.780, 353.171, and 387.211 nm; Y 324.228 nm; Nd 410.945 and 401.224 nm; Pr 410.072 and 417.939 nm; Ca 422.673 nm; Mg 285.213 nm; Na 589.592 and 588.995 nm; Al 396.152 nm; Si 251.432, 251.432, and 251.611 nm; and Fe 238.204 and 259.940 nm.

The concentration of the elements was determined after proper dilution using the calibration curve method (a linear model with a minimum correlation coefficient of 0.999). A series of eight calibration solutions in the concentration range of 0.01–10 mg/L were prepared by appropriate dilution of the standard solutions for this purpose. Ultrapure water (18 MΩ·cm, Simplicity Water Purification Systems, Millipore SAS, Molsheim, France) for the dilution of the standards and samples was utilized. The results were calculated as the average of the concentrations computed for all the used analytical lines.

## 3. Results

The results regarding the adsorption research according to the previously presented procedures for Nd and Y ions on adsorption materials based on modified chitosan are presented below.

**Figure 3:**
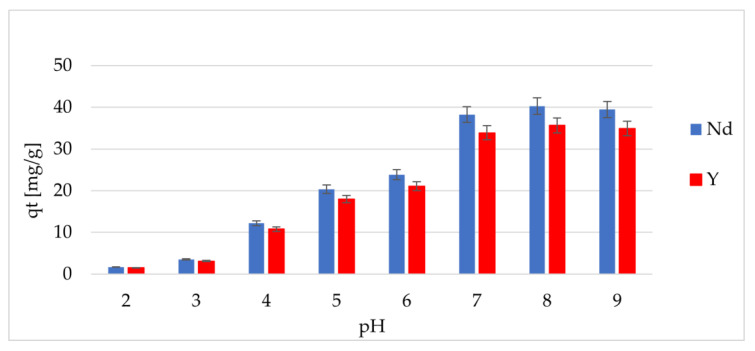
The effect of pH on the adsorption capacity of Nd and Y ions.

Because pH can affect both the physical and chemical behavior of REE ions and the surface properties of adsorbents and their affinity to adsorbed ions, its effect on the adsorption process was investigated (with a pH of 2.0–9.0), as shown in Figure 3. It was found that with the increase of the pH value, the adsorption capacity of the individual REE ions grew. In particular, the adsorption capacity was the highest at a pH of 8.0.

The observed trends are related to the following changes in the considered system. Namely, in a pH range from 2.0 to 4.0, because of the protonation of the amino groups, their binding capacity, consisting of a chelation of the REE ions, is reduced; moreover, the repulsion of the positively charged ions was responsible for the low adsorption capacity.

In a pH range from 4.0 to 7.0, the NH_2_ groups dominated, on which, because of the coordination interactions, the adsorption of REE ions took place. Theoretically, the most optimal pH at which the highest value of the adsorption capacity was reached was a pH of 8.0; however, in a solution with alkaline properties, there is a risk of the precipitation of REE hydroxides. Therefore, a pH of 7.5 was chosen as the optimal pH. It was found that not only the ability to chelate the REE ions but also specific binding sites in the structure of the modified chitosan imprinted with REE ions may be responsible for the increase in the adsorption capacity.

**Figure 4:**
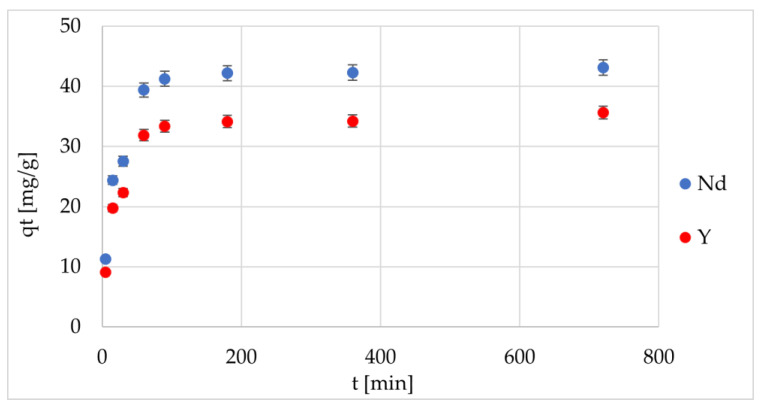
Changes of adsorption capacity over time for two various IIPs.

To explain the mechanisms controlling the course and speed of the adsorption process, kinetic tests (Figure 4) were also conducted. It was found that, for the two types of IIPs, the changes of adsorption capacity in time were characterized by two main stages: namely, a fast initial stage, where over 80% of the REE ions were adsorbed during the first 90 min and a slow second stage lasting until the equilibrium state. As it follows that the adsorption process could be controlled by several mechanisms, such as fast saturation adsorption on the exterior surface of adsorbents, relatively longer contact time to diffuse into the pores of adsorbents to achieve equilibrium and followed by the coordination reaction of functional groups. To analyze the obtained data, the authors used the REE 2.0 and REE_isotherm computer applications specially created for this purpose, and more detailed information about them can be found in a previous publication [43].

Using the first REE 2.0 application, the results were analyzed using several models, including the Lagergren pseudo-first order and pseudo-second order models, the Elovich kinetic model; the intraparticle diffusion model, the diffusion–chemisorption model, and the Boyd model to study the various mechanisms. The obtained results are given in Table 2.

It should be noted that, in all the cases, the experimental data from the adsorption analysis showed a better fit to the pseudo-second order Lagergren model, which indicates the presence of Nd and Y ions in the chemical coordination processes on the IIPs membranes. This was found based on the higher R^2^ values compared to the pseudo-first order model. However, the good fitting of the experimental results to the Elovich model (R^2^ > 0.85) also indicates the existence of chemisorption. In addition, the differences between the values of *q_e_* determined during the simulation and *q_e_* obtained experimentally were significantly smaller for the pseudo-second order Lagergren model. In the case of the intraparticle model, the linear fitting exhibited multilinear curves (R^2^ < 0.61), so two or more mechanisms must be influencing the adsorption process. It was also found that the curves fitted well with the diffusion–chemisorption model (R^2^ > 0.95), because the obtained *q_e_* values were similar to those obtained via the Lagergren pseudo-second order kinetic model, which indicates that the adsorption of REE ions on IIPs can be described using this model. In the case of the Boyd equation, the obtained graphs were non-linear, and none of them passed through the origin, which indicated that the adsorption was significantly controlled by film diffusion. The calculated *D_e_* values corresponded to the values characteristic of chemisorption systems.

**Figure 5:**
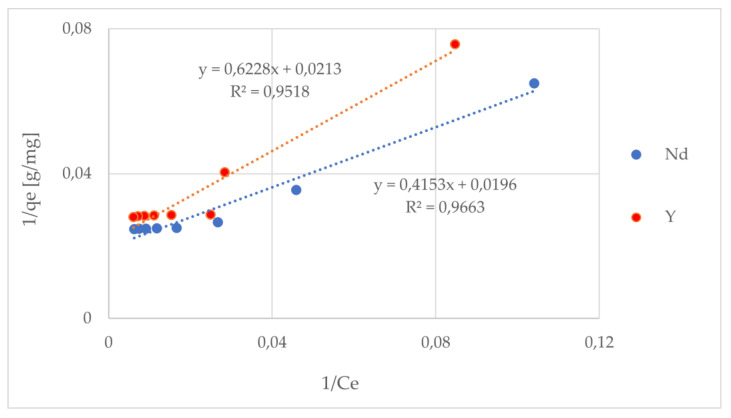
Adsorption equilibrium data and their modeling according to the Langmuir model.

**Figure 6:**
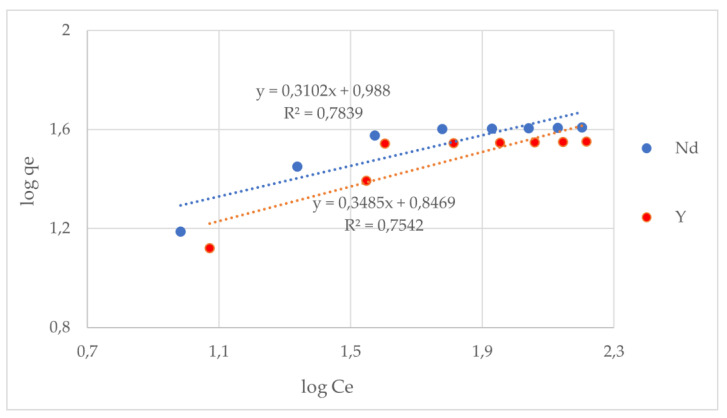
Adsorption equilibrium data and their modeling according to the Freundlich model.

To describe the equilibrium distribution of the REE ions between the liquid and (adsorbent) solid phase for various initial concentrations, adsorption isotherms were determined. They constituted an excellent tool to determine the characteristic parameters, such as the maximum adsorption capacity and the adsorbent affinity for the examined REE ions. It was found that the adsorption capacities increased with the concentration increase until the equilibrium. For the description of the received data, three models—Langmuir, Freundlich, and Dubinin–Radushkevich (Figure 5 and Figure 6)—were used, and the obtained results are presented in Table 3. It was found that a much better fit of the experimental data was obtained with the Langmuir model (R^2^ = 0.966 and 0.952), which indicates the uniform distribution of the binding sites on the adsorbent surface. The adsorption of the Nd and Y ions shows the features of regular monolayer adsorption. The values of the designated adsorption capacity corresponded to those designated from the Langmuir model. The calculated R_L_ values in a range from 0 to 1 indicated the strong affinity of the adsorbents to the examined ions. It should also be noted that the Dubinin–Radushkevich model was a best-fit model to describe the adsorption isotherm data (R^2^ = 0.958 and R^2^ = 0.957). The Dubinin–Radushkevich isotherm model was also used to analyze the adsorption mechanism on a heterogeneous surface (using a Gaussian energy distribution). This model is appropriate to distinguish between the chemical and physical adsorption of metal ions using their free energy per molecule of adsorbate. It is also characteristic of this model to be temperature-dependent. There are three characteristic parameters of this model, namely, *q*m is the maximum uptake of the adsorbate, the Dubinin–Radushkevich isotherm constant is K_DR_ [mol^2^/kJ^2^]; and the mean adsorption energy is E [kJ/mol]. As we can see, the obtained maximum uptakes for both membranes were lower than from the Langmuir model, especially for the Y-imprinted chitosan membrane. The values of K_DR_ were quite similar; however, the mean adsorption energy value of 129.1 [kJ/mol] was higher for the Nd-imprinted chitosan membrane. The values of the mean adsorption energy for both types of membrane were over 120 [kJ/mol] and were, therefore, characteristic for chemisorption (which should be above 80 kJ/mol). As a result, considering the above mentioned data, it could be concluded that the binding sites are uniformly distributed on the sorbent surface, the ion adsorption is monolayer, and the affinity of the sorbent to the separated ions is extremely high.

Selectivity research for three different REEs was performed, namely, Nd and Y, with competitive ions in the form of the five different REE ions and matrix ions that are usually present in the extracts of coal fly ashes.

The selectivity performance was assessed using the K_d_ parameter, i.e., the distribution coefficient and the selectivity coefficients, k (Table 4). It was found that the highest value of K_d_ (1472.8 mL/g) was obtained for the adsorbent substituted with the Nd ions, although other IIPs still had quite high values, exceeding 900 mL/g. This demonstrates a high efficiency in REE recovery using the proposed adsorptive membranes. This may be related to the ligand chelation mechanism and the synergy of the steric effect of imprinting cavities for REE^3+^. Since the amine groups (which are present in the structure of IIPs) belong to the group of hard bases, they have the ability to coordinate the REE^3+^ ions. In addition, the specific size of the imprinting cavities for REE^3+^ created the size selectivity towards Nd^3+^ and Y^3+^ of a certain radius, which is not suitable for the competitive ions.

**Figure 7:**
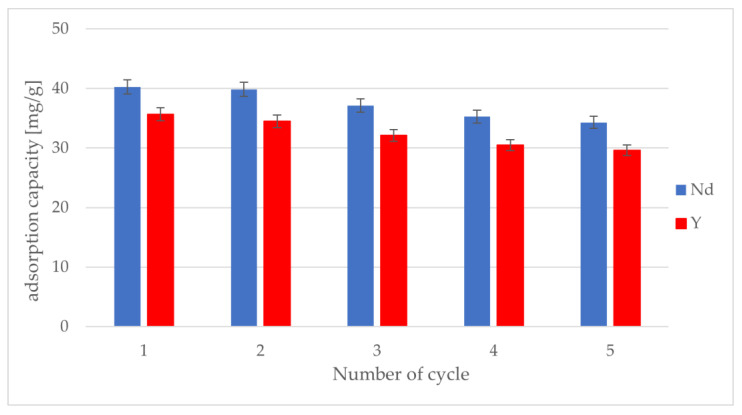
Changes of adsorption capacity during five adsorption–desorption cycles.

Tests were also conducted on the removal and reuse of the tested IIPs. For this purpose, a series of five subsequent adsorption–desorption processes was conducted using a HCl solution, the results of which are shown in Figure 7. A slight decrease occurred for all three IIPs after the first cycle. However, after five cycles, approximately 85% of the value of one cycle was reached. This indicates the possibility of the potential use of synthesized IIPs as adsorbents for the recovery of REEs. In addition, the recovery of REE ions from a solution using the analyzed IIPs was from 71 to 80.5%.

The results for the recovery solution obtained after the separation process performed in the same setup by the application of 1 M of HCl are presented in Table 5, while the concentrations of the various elements in the permeate, retentate, and retention coefficient R are presented in Table 6.

During the analysis of the selective properties of the synthesized membranes, it should be noted (Table 6) that they show a high rejection of the elements that are components of the matrix in the extracts of coal fly ashes, whereas most ions, namely, Na, Mg, Ca, Al, Fe, and Si remain in the retentate, and much smaller values gets into the permeate. On the other hand, these elements, according to the results of the adsorption analysis, do not adsorb in highly selective ion-imprinted membranes and do not appear in the recovered solution (Table 5). However, Nd and Y, for which the ion-imprinted membranes were synthesized, were recovered in amounts of 73.50% and 61.38%, respectively. The retention ratio for these two elements was 90.11% and 80.95%. It should also be noted that the remaining REE ions also undergo partial adsorption in the membrane and constitute a small part of the solution after the recovery. This is, of course, related to the chemical similarity to the reference ions and may be limited by using further modifications of the proposed membranes.

The obtained results testify to the high selectivity of the synthesized membranes in relation to the considered REE ions. At the same time, it should be noted that the membrane intended for the Nd recovery shows a much better adsorption ability. Based on that, it should be stated that these membranes have the potential for further use in the recovery of REEs from coal fly ash extracts and can also be an excellent matrix for hybrid membranes after the introduction of appropriate modifications, which will constitute a further part of the planned works.

**Figure 8:**
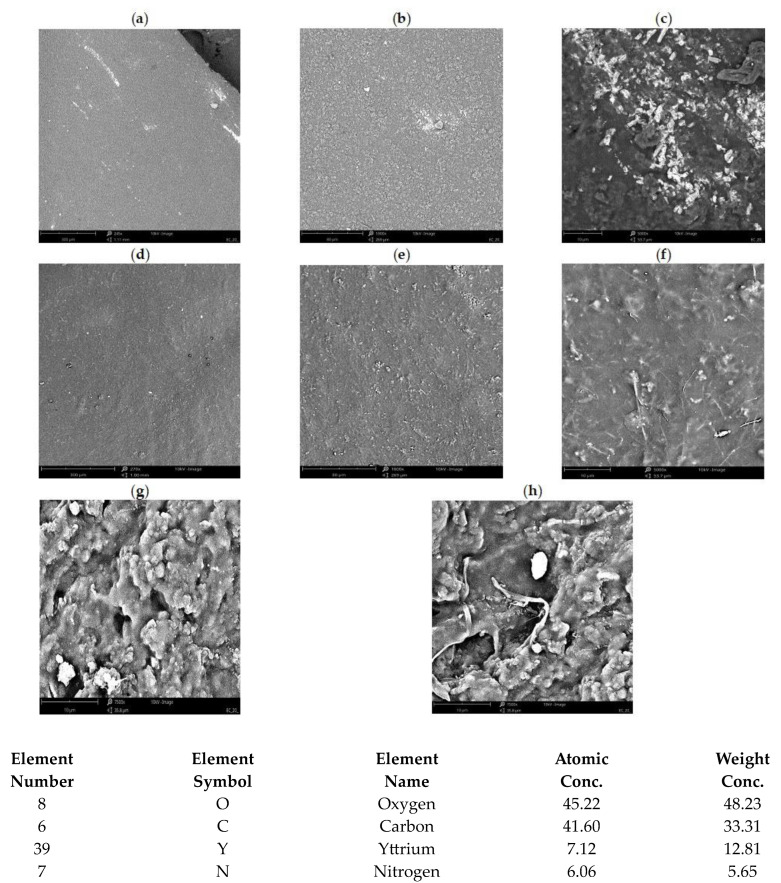
SEM images of the YIIPs membrane with the EDX analysis results for the whole area with (**a**) 245, (**b**) 1000, and (**c**) 5000 magnification; and NdIIPs membrane with (**d**) 270, (**e**) 1000, and (**f**) 5000 magnification. Comparison of SEM images for NdIIPs membrane: (**g**) before and (**h**) after the separation process.

The morphology of the obtained membranes (Figure 8), using the examples of the membranes of YIIPs and NdIIPs, is uniform with small inclusions areas, which are most likely associated with subsequent grains of modified chitosan. The concentration of yttrium ions in a membrane, obtained by the EDX technique, is likely due to the introduced addition of its salt during the synthesis of the membranes. This figure also shows a comparison of the membrane structure, before and after the Nd ion recovery process. It was found that this process did not have a significant negative impact on the structure of these membranes, which may indicate their adequate stability and the possibility of further use in research on the recovery of REE ions from extracts.

**Figure 9:**
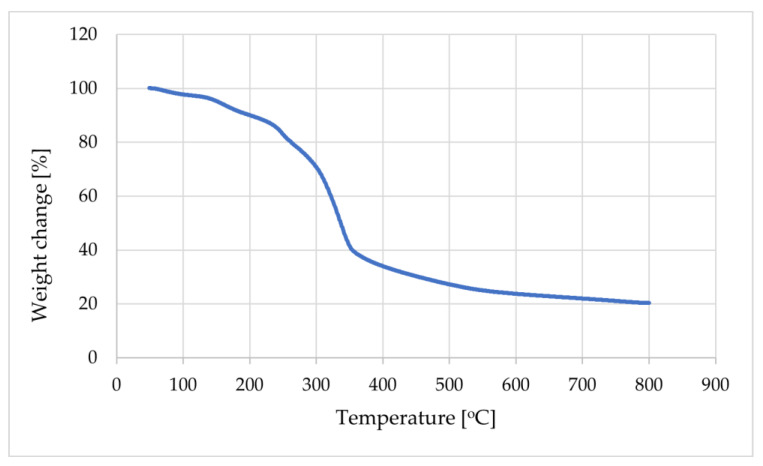
TGA graph of the modified high molecular chitosan and its Schiff base.

The presented curve in Figure 9 contains a few stages, namely, in the range between 50 and 93 °C, where there is a small decrease of about 2%, which could relate to moisture loss. In the next step between 245 °C and 370 °C, a loss of 47% was observed, which could be caused by the loss of the heterocyclic rings and phenyl groups attached to the backbone of chitosan during depolymerization. In the last stage, the weight loss was about 16% and the residual was 20%. That indicated that, until 800 °C, the whole modified chitosan was not decomposed, and the observed loss was caused by decomposition of unreacted amino groups. Comparing the obtained results with the literature results for pure chitosan, it should be noted that after modification the thermal stability of the analyzed material improved (a loss of weight of 11.5% between 27 and 105 °C, 51% between 255 to 363 °C, and 22% in the third stage, with a residual of 8% [41]).

**Figure 10:**
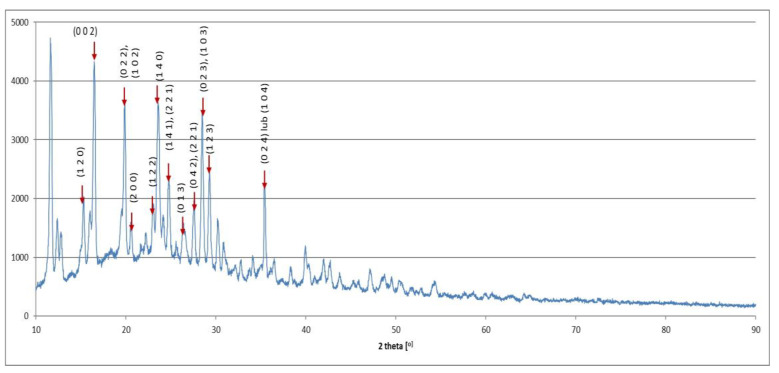
X-ray diffraction spectrum for the high molecular chitosan.

**Figure 11:**
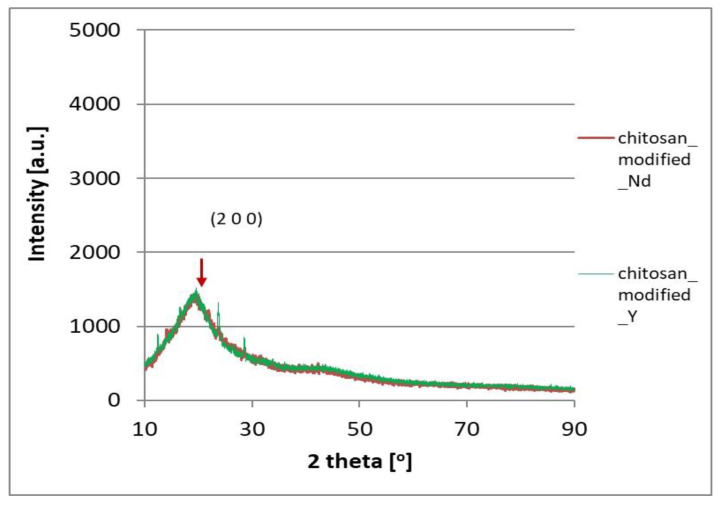
X-ray diffraction spectra for modified high molecular chitosan and Schiff bases substituted with Nd and Y ions.

Figure 10 and Figure 11 present the X-ray diffraction spectra for the high molecular chitosan and its Schiff bases substituted with Nd and Y ions.

The XRD spectra of the high molecular chitosan and its derivatives in the form of Schiff bases substituted with Nd and Y ions were compared (Fig.10 and 11). Some characteristic peaks at 2 θ corresponding to the crystalline lattice planes, such as 10.1^°^ (020), 16.97^°^ (002), 15.18^°^ (120), 20.30^°^ (022 and 102), 21.29^°^ (200 and 040), 23.90^°^ (140 and 220), 25.58^°^ (221), 28.13^°^ (023 and 103), 29.96^°^ (123), and 36.34^°^ (024 and 104) were observed. While for derivatives in the form of Schiff bases substituted with Nd and Y ions the characteristic peaks disappears, becomes wider and weaker, or are shifted. All this may indicate a reduction in the order of the chitosan structure and the Schiff base formation due to the deformation of the strong hydrogen bond in the chitosan backbone through substitution by aldehyde groups on the nitrogen atoms of the chitosan. However, it was found that the Schiff base substituted with Y ions retained a better order in certain directions, with the distances between planes (002), (022), and/or (102), (140), and/or (023), (103) being maintained.

The magnetic properties of the adsorptive membranes are especially important, because of their further influence on the magnetic properties of the prepared hybrid membranes based on the analyzed polymer matrices.

**Figure 12:**
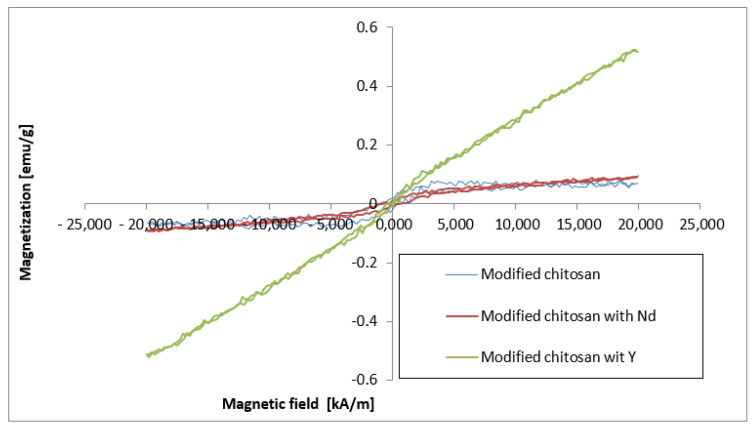
Magnetic hysteresis loops of the adsorptive membranes based on the modified chitosan and the Nd- and Y-imprinted chitosan.

It should be noted that the characteristics of the hysteresis loops of the membranes (Figure 12) based on the modified chitosan and imprinted with the Nd ions are typical for weak ferromagnetic materials. However, in the case of the chitosan imprinted with the Y ions, the shape of the curve and particularly the higher magnetization values with the residual values of coercivity and remanence (Table 7) indicate the transition to superparamagnetic materials.

Table 8 presents a comparison of the adsorption capacity of our synthesized Nd and YIIPs based on the modified chitosan sorbents with other rare earth ion-imprinted adsorbents. It was found that the synthesized IIPs showed a higher or comparable adsorption capacity compared to most of the examples given in the table, although in the case of [44,45] the obtained adsorption capacities were higher. However, this may be directly related to the different forms of occurrence of the tested sorbents, as well as the different conditions of the adsorption process, such as the pH and temperature.

Consequently, the synthesized sorbents have an adequate adsorption capacity, and further work on their development will have research potential in the future. Another advantage of the tested membranes is their harmlessness and ecological potential due to the use of biodegradable chitosan, which can be sustainably recovered at the end of the adsorption process. Therefore, they can be an alternative in the REE recovery processes. The authors intend to use these adsorption materials for further research on hybrid membranes, in which they can constitute an excellent polymer matrix, for example, in membranes with the addition of modified carbon nanotubes with magnetic properties.

## 4. Conclusions

This publication concerns newly synthesized Nd- and Y-selective ion-imprinted membranes based on modified chitosan. Their application in the recovery of selected REEs from synthetic coal fly ash extracts was analyzed. Adsorption tests were performed by considering the impact of pH, kinetics, and isothermal analysis. Selectivity research and research on the reuse of synthesized materials were also performed. In addition, an analysis of the separation abilities of the examined membranes was carried out with the use of the Sterlitech HP4750 high-pressure stirred cell kit. The obtained experimental data were analyzed with two applications, namely, REE 2.0 (including the Lagergren pseudo-first order and pseudo-second order models, the Elovich kinetic model, the intraparticle diffusion model, the diffusion–chemisorption model, and the Boyd model) and REE_isotherm (based on the Langmuir, Freundlich, and Dubinin–Radushkevich models). It was found that the adsorption of Nd and Y ions in the obtained membranes took place according to the chemisorption mechanism and was significantly controlled by film diffusion. The binding sites on the adsorbent surface were uniformly distributed; the adsorbents showed a strong affinity to the examined ions; and the adsorption of the Nd and Y ions showed the features of regular monolayer adsorption. The highest value of K_d_ (1472.8 mL/g) was obtained for the adsorbent imprinted with the Nd ions, although the YIIPs still had quite a high value, exceeding 900 mL/g. This demonstrates a high efficiency in the REE recovery using the proposed adsorptive membranes. After five subsequent adsorption–desorption processes, approximately 85% of the value of one cycle was reached. After modification, the thermal stability of the analyzed materials was improved. The synthesized membranes showed a high rejection of the matrix components (Na, Mg, Ca, Al, Fe, and Si) in the extracts of the coal fly ashes, and the retention ratio for these Nd and Y ions was 90.11% and 80.95%, respectively.

## Figures and Tables

**Table 1:** Composition of feed solution used in tests of the separation abilities of the adsorptive membranes.

REE Ion	Concentration [mg/L]
Nd^3+^	19.13
Pr^3+^	20.00
Dy^3+^	19.27
Gd^3+^	19.41
Y^3+^	19.68
Na^+^	33.56
Mg^2+^	32.33
Ca^2+^	52.95
Al^3+^	104.96
Fe^3+^	56.90
Si^4+^	351.7

**Table 2:** Comparison of the characteristic kinetic parameters from the Lagergren pseudo-first and pseudo-second order models; the Elovich kinetic model; the intraparticle diffusion model; the diffusion–chemisorption model; and the Boyd model.

REE Ion	Model	q_e_ [mg/g]	K_1_ [min^−1^]	K_2_ [g mg^−1^ min^−1^]	a	b	K_p_ [mg g^−1^ min^−1/2^]	x_i_ [mg/g]	K_DC_	D_e_ [m^2^/min]	R^2^
Nd	Pseudo-I order	17.734	0.002								0.499
	Pseudo-II order	40.816		0.002							0.999
	Elovich				6.014	5.561					0.855
	Intraparticle						1.302	14.908			0.610
	Diffusion–chemisorption	49.751							9.842		0.953
	Boyd									2.20 × 10^−12^	0.499
Y	Pseudo-I order	20.814	0.001								0.449
	Pseudo-II order	36.232		0.002							0.999
	Elovich				5.326	4.925					0.855
	Intraparticle						1.153	13.204			0.610
	Diffusion–chemisorption	49.751							9.842		0.953
	Boyd									7.27 × 10^−13^	0.499

**Table 3:** Adsorption equilibrium constants for the Langmuir, Freundlich, and Dubinin–Radushkevich isotherm equations (at 25 °C and a pH of 7.5).

REE Ion	Model	q_m_ [mg/g]	K_L_ [L/mg]	K_F_ [mg/g]	1/n	K_DR_ [mol^2^ kJ^−2]^	E [kJ/mol]	R^2^
Nd	Langmuir	51.020	0.047					0.966
	Freundlich			9.727	0.310			0.784
	Dubinin–Radushkevich	39.411				3.349 × 10^−5^	129.099	0.958
Y	Langmuir	46.948	0.034					0.952
	Freundlich			7.029	0.349			0.754
	Dubinin–Radushkevich	34.898				3.450 × 10^−5^	120.386	0.957

**Table 4:** K_d_ and k values of the analyzed IIPs for a matrix consisting of various metal ions.

REEs	K_d_ [mL/g]	k Nd/Pr	k Nd/Dy	k Nd/Gd	k Nd/Y	k Nd/Na	k Nd/Mg	k Nd/Ca	k Nd/Al	k Nd/Fe	k Nd/Si
Nd	1472.80	22.19	26.31	27.99	31.91	1471.33	524.53	1225.86	667.98	816.75	734.93
Pr	66.37										
Dy	55.99										
Gd	52.61										
Y	46.16										
Na	1.00										
Mg	2.81										
Ca	1.20										
Al	2.20										
Fe	1.80										
Si	2.00										
**REEs**	**K_d_** **[mL/g]**	**k** **Y/Pr**	**k** **Y/Nd**	**k** **Y/Gd**	**k** **Y/Dy**	**k** **Y/Na**	**k** **Y/Mg**	**k** **Y/Ca**	**k** **Y/Al**	**k** **Y/Fe**	**k** **Y/Si**
Y	964.64	16.68	14.48	16.89	17.99	325.50	270.34	518.57	325.37	376.39	349.03
Pr	57.84										
Nd	66.62										
Gd	57.13										
Dy	53.61										
Na	2.96										
Mg	3.57										
Ca	1.86										
Al	2.96										
Fe	2.56										
Si	2.76										

**Table 5:** The recovery of the REE ions from the adsorptive membranes after the separation process.

**REE Ion**	**Recovery from NdIIPs Membrane [%]**	**Recovery from YIIPs Membrane [%]**
Nd	73.50 ± 3.66	5.06 ± 0.27
Pr	4.79 ± 0.23	4.06 ± 0.21
Dy	4.08 ± 0.20	4.31 ± 0.18
Gd	3.84 ± 0.15	3.59 ± 0.16
Y	3.39 ± 0.16	61.38 ± 3.02

**Table 6:** The composition of the permeate and retentate after the separation process for two obtained adsorptive membranes.

Ion	NdIIPs Membrane			YIIPs Membrane		
	Concentration in Permeate [mg/L]	Concentration in Retentate [mg/L]	R [%]	Concentration in Permeate [mg/L]	Concentration in Retentate [mg/L]	R [%]
Na	2.70 ± 0.12	27.25 ± 1.36		4.79 ± 0.22	25.16 ± 1.24	
Mg	2.69 ± 0.14	27.20 ± 1.41		4.78 ± 0.25	25.11 ± 1.29	
Ca	4.49 ± 0.22	45.41 ± 2.23		7.98 ± 0.39	41.92 ± 2.02	
Al	8.79 ± 0.41	88.92 ± 4.56		15.63 ± 0.77	82.08 ± 4.11	
Fe	4.76 ± 0.23	48.11 ± 2.44		8.46 ± 0.42	44.41 ± 2.31	
Si	26.98 ± 1.45	272.81 ± 13.26		47.87 ± 2.42	251.32 ± 12.99	
Nd	0.48 ± 0.03	4.82 ± 0.24	90.11	3.04 ± 0.15	15.95 ± 0.81	
Pr	1.71 ± 0.08	17.33 ± 0.86		3.07 ± 0.13	16.12 ± 0.86	
Dy	1.73 ± 0.07	17.46 ± 0.81		3.06 ± 0.12	16.08 ± 0.77	
Gd	1.73 ± 0.08	17.50 ± 0.79		3.09 ± 0.16	16.20 ± 0.83	
Y	1.74 ± 0.09	17.58 ± 0.89		1.24 ± 0.07	6.49 ± 0.39	80.95

**Table 7:** Magnetic properties of the obtained membranes.

Membrane	Saturation Magnetization [emu/g]	Remanence [emu/g]	Coercivity [kA/m]
Modified chitosan	0.085	0.0194	549.0
Nd-imprinted chitosan	0.094	0.0074	736.4
Y-imprinted chitosan	0.5235	0.0057	53.2

**Table 8:** Comparison of the adsorption properties with other sorbents.

Sorbent	Cation	Adsorption Capacity [mg/g]	References
IMCFs	Nd(III)	22.61	[46]
Dual-template docking oriented ionic imprinted mesoporous films	Nd(III)	34.98	[14]
Ionic imprinted polymer particles	Nd(III)	33	[47]
Phosphonic acid functionalized silica microspheres	Nd(III)	45	[45]
Molecularly imprinted polymers	Nd(III)	14.6	[48]
Ion-imprinted mesoporous silica materials	Dy(III)	22.3	[49]
Diethylenetriamine-functionalized chitosan magnetic nanobased particles	Dy(III)	51.5	[50]
Magnetic chitosan nanobased particles grafted with amino acids	Dy(III)	8.9–17.6	[51]
II-MAC	Dy(III)	23.3	[15]
Imprinted cryogel using N-methacryloamido antipyrine as functional monomer	Ce(III)	36.58	[22]
Imprinted polymer using Schiff base	Ce(III)	24.5	[32]
Imprinted polymer using 4-(2,4-dihydroxyphenazylo)acetophenone	Ce(III)	24.7	[52]
Imprinted styrene-divinylebenzene copolymer	Dy(III)	40.15	[25]
Ionic imprinted resins based on EDTA and DTPA derivatives	Gd(III)	24.53	[53]
IIP-HQP/SiO_2_	Pr(III)	18.32	[54]
Sc(III) ion-imprinted polymers	Sc(III)	12.8	[55]
NdIIPs based on modified chitosan	Nd(III)	40.8	This work
YIIPs based on modified chitosan	Y(III)	36.2	This work

## Data Availability

The raw data supporting the conclusions of this article will be made available by the authors on request.
